# An imaging-based approach predicts clinical outcomes in prostate cancer through a novel support vector machine classification

**DOI:** 10.18632/oncotarget.11293

**Published:** 2016-08-15

**Authors:** Yu-Dong Zhang, Jing Wang, Chen-Jiang Wu, Mei-Ling Bao, Hai Li, Xiao-Ning Wang, Jun Tao, Hai-Bin Shi

**Affiliations:** ^1^ Department of Radiology, the First Affiliated Hospital with Nanjing Medical University, Nanjing, China; ^2^ Center for Medical Device Evaluation, CFDA, Beijing, China; ^3^ Department of Pathology, the First Affiliated Hospital with Nanjing Medical University, Nanjing, China; ^4^ Department of Urology, the First Affiliated Hospital with Nanjing Medical University, Nanjing, China

**Keywords:** prostate cancer, biochemical recurrence, MRI, radical prostatectomy, support vector machine

## Abstract

Preoperatively predict the probability of Prostate cancer (PCa) biochemical recurrence (BCR) is of definite clinical relevance. The purpose of this study was to develop an imaging-based approach in the prediction of 3-years BCR through a novel support vector machine (SVM) classification. We collected clinicopathologic and MR imaging datasets in 205 patients pathologically confirmed PCa after radical prostatectomy. Univariable and multivariable analyses were used to assess the association between MR findings and 3-years BCR, and modeled the imaging variables and follow-up data to predict 3-year PCa BCR using SVM analysis. The performance of SVM was compared with conventional Logistic regression (LR) and D'Amico risk stratification scheme by area under the receiver operating characteristic curve (Az) analysis. We found that SVM had significantly higher Az (0.959 vs. 0.886; *p* = 0.007), sensitivity (93.3% vs. 83.3%; *p* = 0.025), specificity (91.7% vs. 77.2%; *p* = 0.009) and accuracy (92.2% vs. 79.0%; *p* = 0.006) than LR analysis. Performance of popularized D'Amico scheme was effectively improved by adding MRI-derived variables (Az: 0.970 vs. 0.859, *p* < 0.001; sensitivity: 91.7% vs. 86.7%, *p* = 0.031; specificity: 94.5% vs. 78.6%, *p* = 0.001; and accuracy: 93.7% vs. 81.0%, *p* = 0.007). Additionally, beside pathological Gleason score (hazard ratio [HR] = 1.560, *p* = 0.008), surgical-T3b (HR = 4.525, *p* < 0.001) and positive surgical margin (HR = 1.314, *p* = 0.007), apparent diffusion coefficient (HR = 0.149, *p* = 0.035) was the only independent imaging predictor of time to PSA failure. Therefore, We concluded that imaging-based approach using SVM was superior to LR analysis in predicting PCa outcome. Adding MR variables improved the performance of D'Amico scheme.

## INTRODUCTION

Radical prostatectomy (RP) is an effective form of local therapy for prostate cancer (PCa) [1, 2]. However, there is still approximately one quarter of patients undergoing this curative surgery will have a biochemical recurrence (BCR) or “prostate-specific antigen (PSA) recurrence” [1-5]. Preoperatively predict the probability of BCR is of definite clinical relevance. Several preoperative nomograms, i.e., the Stephenson nomogram [6], the D'Amico risk stratification scheme [7], and the University of California, San Francisco, Cancer of the Prostate Risk Assessment (CAPRA) score [8], have been developed in the urologic community to predict the probability of BCR within 3-5 or 10 years of treatment. Although these nomograms have been internationally validated, unfortunately, only a few of them have predicted the probability of 5-year BCR with more than 70% accuracy [9-11]. Therefore, efforts to improve existing outcome prediction tools in PCa are always encouraged.

In the past decades, multi-parametric MRI (mp-MRI), i.e., T1-weighted imaging (T_1_WI), T2-weighted imaging (T_2_WI), diffusion weighted imaging (DWI), dynamic contrast-enhanced (DCE), and MR spectroscopic imaging, has been investigated as promising way not only for imaging, detecting and staging the localized cancer, but also for risk stratification, image guidance for biopsy, surgery and focal therapy [12-18]. However, the clinical application of mp-MRI can be challenged by large amount of image data in each patient. Additionally, as without standardized ways, the image interpretation can be subjective, which depends on radiologists' experience, level of local medical culture and personal preference, thereby limiting the diagnostic accuracy and reproducibility in PCa [19]. Recently, automated cancer detecting and classification based on preoperative mp-MRI through a novel support vector machine (SVM) analysis has been an ongoing interest. SVM is one of the best-known classification techniques and usually provides the best classification for computer-aided detection in radiology [20]. Recent technical developments in SVM by expending advanced algorithms (e.g., principal component analysis [21, 22], particle swarm optimization [23] and cross-validation schemes) have produced encouraging results regarding solving or classifying pattern recognition problems [24]. The SVM analysis has the ability of reducing false positives in the determination of abnormal lesions in brain [24, 25], breast [26] and prostate [20], showing high accuracy, elegant mathematical tractability and direct geometric interpretation. Although the application of SVM to the diagnosis of PCa has validity in research, a more meaningful clinical application of it is in helping the identification of predictors of outcome [9, 27, 28]. These could help direct, to more high-risk individuals, the early implementation of targeted interventions that have been shown to reduce relapse rates, such as optimized neo-adjuvant therapy, resulting in better clinical outcomes. However, there is not yet a single, widely accepted algorithm for establishing that predictive nomogram by mp-MRI.

The purpose of this study was therefore: (1) to develop an imaging-based nomogram to predict 3-years BCR in patients with localized PCa after RP using SVM analysis; (2) to determine whether SVM could be better than a conventional logistic regression (LR) analysis to improve performance ability; (3) to evaluate whether adding MRI-derived variables can effectively complement the preoperative clinico-pathologic predictors in patient outcome predicting.

## RESULTS

### Clinical characteristics

Clinical, histologic variables and MR findings for the study cohort (205 patients) are depicted in Table 1. The median age was 68 yr (interquartile range [IQR]: 62- 73 ys), and median serum PSA was 13.1 ng/ml (IQR: 7.9- 17.7). As of January 2016, 61 (29.7%) of the 205 patients in the study had a biochemical recurrence. The median (range) follow-up for all patients was 43.8 (2-60) months; it was 47.3 (37-60) months for those with no evidence of recurrence and 20.1 (2-41) months for those with recurrence. Of the 205 prostatectomy confirmed patients, total 409 lesions were detected at histological findings, whereas total 263 lesions were detected at MR findings. Tumor in 178 (86.8%) patients originated in PZ and 27 (13.2%) originated in TZ on section histology.

**Table 1:** Clinical, pathological and MR findings of cohort (n = 205)

Variables	Mean (SD) or n (%)	
Preoperative Characteristics		
Age, n (%)		
≤ 60 ys	40 (19.5%)	
> 60 ys	165 (80.5%)	
Clinical stage, n (%)		
< T3	118 (57.6%)	
≥ T3	87 (42.4%)	
Preoperative PSA level, n (%)		
0-10 ng/ml	81 (39.5%)	
10-20 ng/ml	100 (48.8%)	
> 20 ng/ml	24 (11.7%)	
Biopsy Gleason score, n (%)		
≤ 3+3	88 (42.9%)	
3+4	55 (26.8%)	
4+3	44 (21.5%)	
≥ 4+4	18 (8.8%)	
Preoperative MR findings		
Lesion Location, n (%)		
PZ	178 (86.8%)	
TZ	27 (13.2%)	
DCE type		
Type 1+2	86 (41.9%)	
Type 3	119 (58.0%)	
ADCs (× 10^−3^ mm^2^/s), Mean (SD)	1.06 (0.19)	
Max diameter (cm), Mean (SD)	1.8 (0.8)	
MR-detected T stage		
< T3a	114 (55.6%)	
T3a (ECE)	56 (27.3%)	
T3b (SVI)	35 (17.1%)	
Postoperative Characteristics		
Pathological Gleason score, n (%)		
≤ 3+3	48 (23.4%)	
3+4	75 (36.6%)	
4+3	48 (23.4%)	
≥ 4+4	34 (16.6%)	
Surgical tumor volume, Mean (SD)		
Volume, cm^3^	5.4 (9.1)	
Volume percentage, %	14.5 (14.6)	
Surgical ECE, n (%)		
Absent	133 (64.9%)	
Present	72 (35.1%)	
Surgical SVI, n (%)		
Absent	166 (81.0%)	
Present	39 (19.0%)	
Cancer invasion on surgical margin, n (%)		
Negative	154 (75.1%)	
Positive	51 (24.9%)	

PSA = prostate-specific antigen; PZ = peripheral zone; TZ = transition zone; DCE = dynamic contrast-enhanced; ADC = apparent diffusion coefficient; ECE = extracapsular extension; SVI = seminal vesicle invasion.

**Figure 1:**
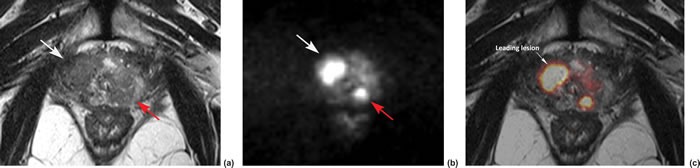
MR images of a representative case to indicate the imaging registration, lesion identification and region of interesting drawing A 69-year-old man presented with two solid tumor foci in right peripheral zone (PZ; write arrow) (pathological Gleason score 4+4, pT3a) and left PZ (pathological Gleason score 4+3), respectively. The two lesions were characterized with hypo-signal intensity (SI) on T2-weighted imaging (T2WI; a), and hyper-SI on diffusion-weighted imaging (DWI; b = 1000 s/mm^2^). Registration and fusion of images between T2WI and DWI was performed using **a.** DICOM-tag metrics **c.**, showing clearly the lesion boundaries. Because the lesion in right TZ had larger size, and an infiltration of the peri-prostatic fat (extracapsular extension) was suspected, it was selected as leading lesion for further quantitative measure.

### Performance of the predictive models

Table 2 shows the results of the multiple LR analysis. The estimate probabilities (*Pi*) of PCa BCR after RP is: *Pi* = 1 / (1 + exp(−0.421 + 1.226 * age + 1.211 * MR T-stage - 0.170 *Dmax + 0.645 * MR visible - 0.150 * location - 4.196 * ADCs + 0.407 * DCE type + 0.266* PI-RADS)). It shows that tumor ADCs and MR T-stage are significantly associated with 3-years BCR (*p* < 0.05).

**Table 2:** Logistic regression (LR) model with multiple variables

Variable	Coefficient B	S.E.	Wald	*p*	Exp(B)	95% C.I. for EXP(B)	
						Lower	Upper
DCE type	0.407	0.443	0.743	0.359	1.502	0.630	3.579
ADCs	−4.196	1.346	9.722	0.002*	0.015	0.001	0.210
Location	−0.150	0.567	0.069	0.792	0.861	0.283	2.618
MR visible	0.645	1.172	0.303	0.582	1.906	0.191	18.974
PI-RADS	0.266	0.403	0.434	0.510	1.304	0.592	2.874
Dmax	−0.170	0.280	0.369	0.543	0.844	0.488	1.459
MR T-stage	1.211	0.299	16.397	0.001*	3.358	1.868	6.037
Age	1.226	0.643	3.637	0.057	3.406	0.967	12.002
Constant	−0.421	2.259	0.035	0.852	0.656		

C.I. = Confidence Interval, S.E. = Standard Error. PI-RADS = Prostate Imaging and Reporting and Data System.

Figure 3 shows the ROC curves for the four risk predictive models. The area under the ROC curve values (Az) for the model of LR_MR_, SVM_MR_, SVM_D'Amico_, and SVM_D'Amico+MR_ was 0.886, 0.959, 0.859, and 0.970, respectively. Pairwise comparison of the ROC curves demonstrated that there was statistical difference between LR_MR_ and SVM_MR_ (Figure 4a; *p* = 0.007), and between SVM_D'Amico_ and SVM_D'Amico+MR_ (Figure 4b; *p* < 0.001). Using the optimal cutoff values obtained from ROC analysis (Table 3), SVM_MR_ had significantly higher diagnostic SEN (*p* = 0.025), SPE (*p* = 0.009) and ACC (*p* = 0.006) than LR_MR_ in 3-years BCR predicting. SVM_D'Amico+MR_ had significantly higher diagnostic SEN (*p* = 0.031), SPE (*p* = 0.001) and ACC (*p* = 0.007) than SVM_D'Amico_. The difference of Az, SEN, SPE and ACC between SVM_MR_ and SVM_D'Amico+MR_ was insignificant (all *p* > 0.05). Figure 4 shows the predicted BCR-free survival curves of 205 patients by LR_MR_, SVM_MR_, SVM_D'Amico_ and SVM_D'Amico+MR_ model, respectively, which were compared to patients' true survival curve. It shows that the survival functions constructed by SVM_MR_ and SVM_D'Amico+MR_ have relatively smaller bias than LR_MR_ and SVM_D'Amico_.

**Table 3:** Predictabilities of LR, ANN and SVM models in prediction of PCa BCR

Model	Az	SEN, %	SPE, %	ACC, %	cutoff value
LR_MR_	0.886 (0.834-0.926)	83.3 (71.5-91.7)	77.2 (69.5-83.8)	79.0 (70.1-86.2)	Pi > 0.41
SVM_MR_	0.959 (0.922-0.982)	93.3 (83.8-98.1)	91.7 (86.0-95.6)	92.2 (85.3-96.3)	Pi > 0.44
SVM_D'Amico_	0.859 (0.804-0.903)	86.7 (75.4-94.0)	78.6 (71.0-85.0)	81.0 (72.3-87.7)	Pi > 0.41
SVM_D'Amico+MR_	0.970 (0.936-0.988)	91.7 (81.6-97.2)	94.5 (89.4-97.6)	93.7 (87.1-97.4)	Pi > 0.40

Note.-data in brackets are 95% C.I.; LR, logistic regression; SVM, support vector machine; Az, areas under the ROC curve, SEN, sensitivity; SPE, specificity; ACC, accuracy.

**Figure 2:**
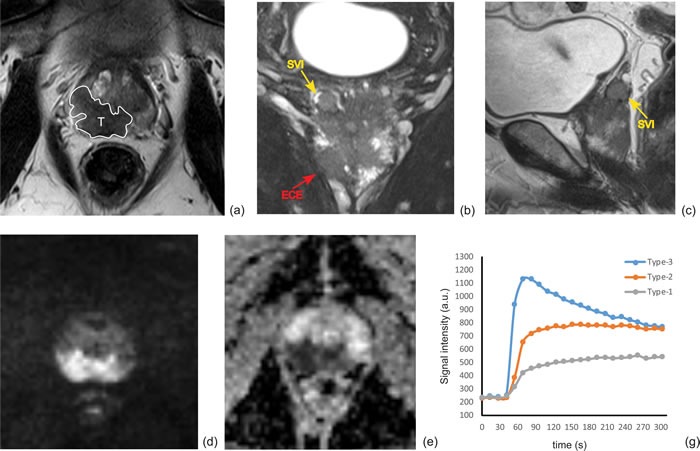
A multi-parametric prostate MRI in a 68-year-old man (PSA of 34.3 ng/ml, biopsy Gleason score 4+3 and stage T3b) to show the metrics for imaging interpretation **a.** Tumor featured with decreased SI on axial T_2_WI, the location was defined at PZ (white line). **b.** ECE (red arrow) and seminal vesicle invasion (SVI; yellow arrow) were notified on coronal T_2_WI with fat suppression. **c.** Tumor with SVI was confirmed on sagittal T_2_WI (yellow arrow). **d.** Tumor was characteristic with hyper-SI on DWI (b = 1000 s/mm^2^) and decreased ADC **e. f.** Schematic diagram shows three-type DCE curves, for this patient, tumor was defined as type-3 DCE curve (blue color). Histopathologic results showed a pathological Gleason core 4+4 and surgical SVI in this patient, and PSA failure was determined on 16 months after the prostatectomy treatment.

**Figure 3:**
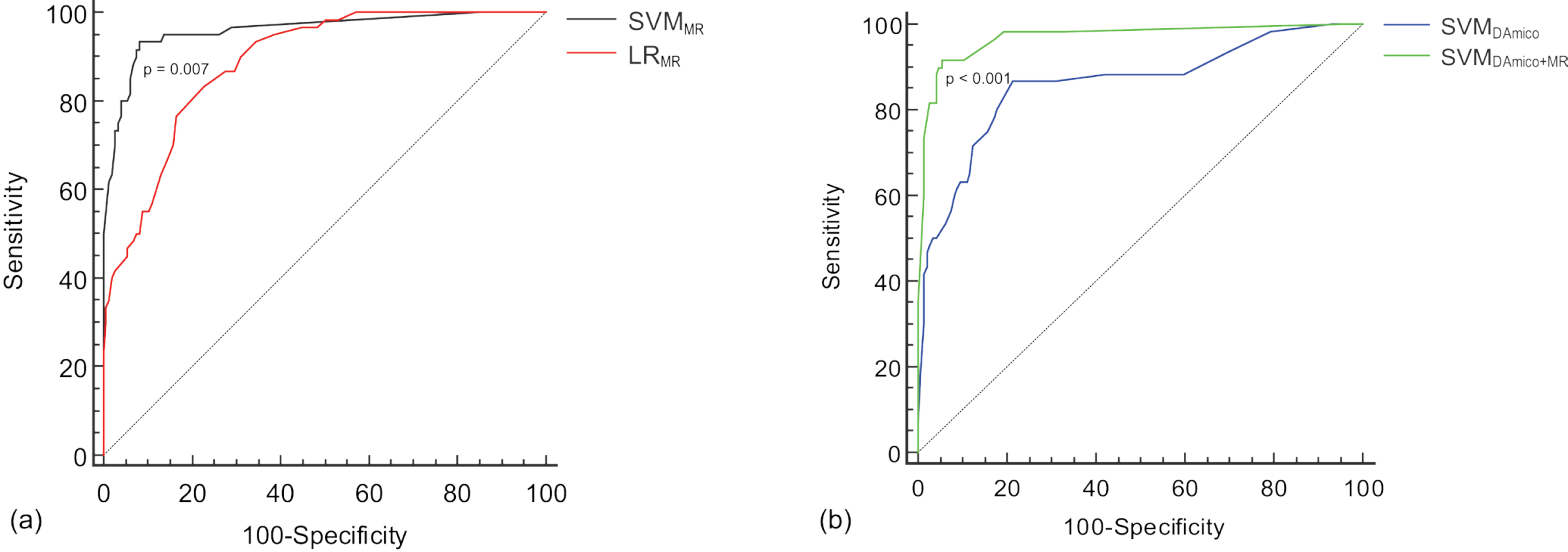
the comparison of ROC curves among four risk predictive models constructed with different classification methods and input variables **a.** with the same MR input variables, the model constructed by support vector machine (SVM_MR_) has significantly higher area under the ROC curve value (Az = 0.959) than the model of logistic regression (Az = 0.886, p = 0.007). **b.** using the same SVM analysis, the model combining MR and DA'mico variables has significantly higher Az (0.970) than the model using sole DA'mico variables (Az = 0.859; *p* < 0.001).

**Figure 4:**
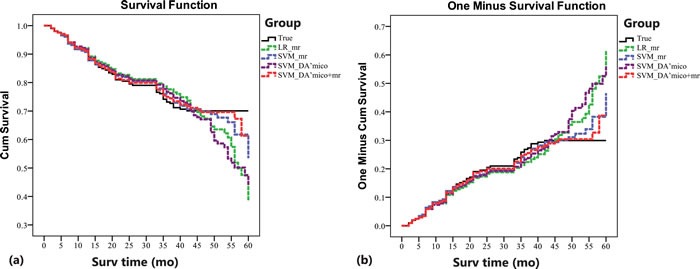
Predicted BCR-free Kaplan-Meier curves of 205 patients after radical prostatectomy by four constructed models The curves are stratified by: patients' true Kaplan-Meier curve (black), and predicted Kaplan-Meier curves by LR_MR_ (green), SVM_MR_ (blue), SVM_D'Amico_ (brown) and SVM_D'Amico+MR_ model (red), respectively. It shows that the Kaplan-Meier functions constructed by SVM_MR_ and SVM_D'Amico+MR_ have relatively smaller bias than these of LR_MR_ and SVM_D'Amico_.

Among all the preoperative clinic-pathologic variables (age, biopsy GS, clinical TNM stage, and baseline PSA), the MR variables (tumor location, MR-visible, Dmax, DCE type, ADCs, PI-RADS score, and MR T-stage), and the postoperative variables (tumor volume, volume percentage, pathological GS, pathological T-stage, cancer invasion on surgical margin, and perineural invasion), the Cox regression multivariate analysis indicated that only the tumor ADCs (HR = 0.149; *p* = 0.035), pathological GS (HR = 1.560; *p* = 0.008), positive surgical margin (HR = 2.314; *p* = 0.007), and surgical SVI (HR = 4.525; *p* < 0.001) were the independent risk predictors of time to PSA failure (Table 4). Using the optimal cutoff values for ADCs obtained from ROC analysis (< 1.03 × 10^−3^ mm^2^/s), The overall accuracy of ADCs to differentiate low-intermediate grade (pathological GS ≤ 3+4) from high-grade GS > 3+4) PCa is 64.9%. The overall accuracy of MR to determine ECE and SVI is 81.9% and 95.1%, respectively.

**Table 4:** Cox regression univariate and multivariate predictors of BCR 3 years after RP treatment (preoperative clinic-pathologic, MR, and postoperative findings)

Variable	Kaplan-Meier analysis		multivariate Cox analysis		
	Log-rank (Mantel-Cox)	*p*	B	HR (95% CI)	*p*
preoperative clinic-pathologic variables					
Age	8.691	0.003			
PSA level	108.833	< 0.001			
Biopsy GS	79.987	< 0.001			
Clinical stage	19.963	< 0.001			
Preoperative MR variables					
Tumor location	0.445	0.505			
MR-visible	10.558	0.001			
PI-RADS	18.366	< 0.001			
Dmax	13.663	< 0.001			
DCE type	14.015	< 0.001			
ADCs	42.122	< 0.001	−1.904	0.149 (0.025- 0.873)	0.035^†^
MR T stage	90.668	< 0.001			
postoperative variables					
Volume	12.153	< 0.001			
Volume percentage	12.938	< 0.001			
Pathological GS	71.625	< 0.001	0.445	1.560 (1.124-2.166)	0.008^†^
Surgical T-stage	128.576	< 0.001	1.510	4.525 (2.441-8.386)	< 0.001^†^
Surgical margin	70.056	< 0.001	0.839	2.314 (1.260-4.251)	0.007^†^
Perineural invasion	11.632	0.001			

Note. -PSA = prostate-specific antigen; GS = Gleason score; DCE = dynamic contrast-enhanced; ADC = apparent diffusion coefficient; HR = hazard ratio.

† significant correlates tested by Cox regression multivariate analysis.

## DISCUSSION

In this study, we demonstrated that the combination of clinicopathologic and imaging variables can effectively contribute to the prediction of clinical outcome in patients with localized PCa before the surgical treatment. The nomogram constructed by SVM was significantly better than LR analysis. Additionally, the performance of popularized D'Amico scheme can be effectively improved by adding mp-MR imaging markers, suggesting this approach could be an optimal tool for initial evaluation before a curative attempt in patients with localized PCa.

The classification methods applied to pelvic MR image data was initially investigated in several studies for automated detection of malignancy [29-31]. Poulakis devised an ANN model with the input variables of MR findings, pretreatment PSA, clinical TNM stage and biopsy GS to predict PCa recurrence in 210 clinically localized PCa [9]. They found the new nomogram can produce higher sensitivity (91%) and specificity (88%) than conventional LR analysis to preoperatively predict 5-year PSA failure. Fuchsjäger et al. [32] explored an integrated Cox model based on a seven-point scale MR scoring system and clinical variables to preoperatively predict 5- and 10-year BCR after RP. And recently, Park et al. [33, 34] demonstrated that tumor ADCs derived from DWI and new PI-RADS v2 score was better than preoperative PSA, biopsy GS and surgical variables in the prediction of PSA failure in 158 consecutive cases. These nomograms can be competitive to the available clinical nomograms such as the D'Amico risk scheme [7], the CAPRA score [8], and the Stephenson nomogram [6].

Our study improved the predictive performance by applying a robust SVM analysis with MR and clinic-pathologic variables. The SVM produced a 93.3% SEN, 91.7% SPE and 92.2% ACC, showing significantly better performance ability than LR approach. This can be attributed to the fact that SVM classifier depends only on the support vectors, and the classifier function is not influenced by the entire data set. It can progressively learns from misclassified examples and automatically remove the false positives *via* examination of the distance in the Hilbert space to avoid over-fitting. Second, RBF-SVM uses a cost-factor to control for classifier complexity and the prediction accuracy which would decrease in case of small cost-factor. Although the data presented in this paper were based on an optimal cost-factor of 2.934, we tested a range of different cost-factor values from 0.1 to 50 and found very similar results, suggesting its good reproducibility. Third, different to the nomograms reported by Poulakis [9], Fuchsjäger [32] and Park [33], our study included more new imaging markers, i.e., DCE-type, Dmax, ADCs and MR T-stage, which may contributed to the improvement of predictive performance. This is true as the performance of clinical D'Amico scheme can be efficiently increased by adding these imaging markers. The imaging contribution in PCa BCR predicting can be explained that, the newly proposed imaging markers, i.e., ADCs, DCE type, MR T-stage, might indicate the postoperative histopathological features of PCa more accurately than conventional clinical variables. This is true as the quantitative MR imaging parameters, e.g., DCE-derived Ktrans, DWI-derived ADCs and/or Dapp, had been identified to be highly associated with the localized cancer aggressiveness and prognosis in recent clinical studies [16-18, 33, 35]. Additionally, we found that, among all imaging markers, only tumor ADCs was independently related to 3-years BCR, suggesting its prognostic value in the prediction of clinical outcome in PCa. The overall ACC for ADCs to differentiate low/intermediate- from high-grade PCa is 64.9%, partly consistent with previous reports [16, 18, 36]. And our mp-MRI produced high accuracy for detecting surgical ECE (81.9%) and SVI (95.1%). This result confirms strongly the thesis that MR findings can complement the clinical nomogram for the improvement of the predictive performance. The nomogram by integrating MR findings and preoperative clinical variables such as D'Amico risk score is noninvasive and prospective, which could be readily applied by clinicians and allows for immediate identification of the variables accounted for BCR before the RP.

An important limitation is the relatively small sample size in this preliminary study. Thus, future work should consider validating the accuracy of our classifier with an independent larger sample of patients. Second, part of input variables, e.g., DCE-type, MR visibility and MR T-stage, was generally interviewed by individual radiologists, the results of which, to some extent, depending on radiologist's experience. Deviation is unavoidable in spite of many efforts to make. Thus, the findings under this calculation should be considered with caution. Finally, as this study used data from a single medical center, it remains unclear to what extent differences in acquisition protocol or scanners or patient cohort affect the accuracy of the classifier.

## CONCLUSIONS

A SVM approach was more accurate than the classical LR analysis with the same input variables in the prediction of 3-years BCR in PCa after RP. Additionally, MR parameters can efficiently improve the predictive performance of popularized D'Amico scheme. This confirms the thesis that MR findings can well complement the preoperative clinic-pathologic variables to improve existing outcome prediction tools in PCa. As this machine learning approach is benefited with no user input, optimal reproducibility, faster post-processing times, and the ability to use the full potential of the combined clinical and diagnostic variables, which could be readily applied by clinicians to preoperatively assess the therapeutic risk.

## MATERIALS AND METHODS

### Patients

Our local institutional review board approved and waived the informed consent requirement for this retrospective study. Between January 2009 and February 2013, 295 consecutive patients with biopsy confirmed PCa underwent prostatic MRI before RP. Patients received postoperatively immediate adjuvant hormone or radiation therapy before a documented decease recurrence (*n* = 23, 7.8%), patients who were lost from follow-up (*n* = 19, 6.4%), and patients failed to receive a standardized MR examination (*n* = 21, 7.1%) or underwent the MR examination from outside institutions (*n* = 27, 9.2%) were excluded from the study. Thus, 205 patients were eligible for clinical evaluation. No patient received any neo-adjuvant therapy. The median time interval between the MR examination and prostatectomy was 12 days (range, 5-17 days).

### Prostatic MR examination

All imaging examinations were performed with 3.0-T MR scanners (Trio and Verio Tim; Siemens, Erlangen, Germany) and pelvic phased-array coils. As per the standard prostatic MRI at our institution, the images obtained included transverse T_1_-weighted turbo spin-echo (TSE) images (TR/TE, 700/14 ms; section thickness, 3.5 mm; intersection gap, 0.3 mm; field of view, 25 cm; and matrix, 384 × 336) and transverse, coronal, and sagittal T2-weighted TSE images (TR/TE, 6000/124 ms; section thickness, 3.5 mm; intersection gap, 0.3 mm; field of view, 25 cm; and matrix, 384 × 336) of the prostate and seminal vesicles. Then, single-shot echo-planar imaging (TR/TE, 6800/98 ms; field of view, 25 cm; matrix, 192 × 130; section thickness, 3.5 mm; intersection gap, 0.3 mm; and a parallel imaging factor of 2) was performed with diffusion-module and fat suppression pulses. Diffusion in three directions was measured by using b values of 0, 50, 150, 300, 600, and 1000 s/mm^2^. After a routine MR examination, a T1-weighted gradient recalled echo sequence was prescribed to acquire DCE-MR imaging data. This protocol was performed with parameters as follows: TR/TE, 3.8/1.8; flip angle, 12°; field of view, 36 cm; matrix, 384 × 384; slice thickness, 3.5 mm; intersection gap, 0.3 mm). After two acquisitions, a bolus of Gd-diethylenetriaminepenta-acetic acid (Gd-DTPA 0.05 mmol/kg; Magnevist, Bayer AG, Berlin, Germany) was injected at a rate of 2.5 ml/s through a 20-gauge antecubitalintravenous line. Bolus injection was performed with a MR-compatible power injector (Spectris; Medrad, Pittsburgh, PA) followed by a 15-ml saline flush. The DCE-MR imaging was continued for 5.0 minutes after the Gd-DTPA injection.

### Imaging analysis

Imaging analysis was completed independently by two experienced radiologists (Y.Z. and X.W. with 6 and more than 20 years of experience in reading prostate MR imaging). Because this study is to determine whether the noninvasive mp-MRI has prospective value in predicting BCR before the RP operation, any histologic-radiologic correlation was prohibited during the MR imaging analysis. In this procedure, acquisition date and participant identification were removed from all images. The investigators were blinded to all clinical information. The mp-MR images from axial T_2_WI, DWI and DCE were registered by expending a DICOM-tags metrics on a computer-aided platform (FireVoxel; Center for Advanced Imaging Innovation and Research [CAI^2^R], New York University School of Medicine, New York, NY). The fusion images were displayed simultaneously and explored slice by slice to facilitate lesion discovery and region of interest (ROI) drawing. In each prostate, the radiologists first identified the most suspicious cancer lesion (leading lesion) on peripheral zone (PZ) and/or transition zone (TZ). The observation featured with largest lesion size, and/or dominantly low signal intensity (SI) on apparent diffusion coefficient (ADC) maps, and/or suspected extracapsular extension (ECE), and/or suspected seminal vesicle invasion (SVI), was defined as the leading lesion (Figure 1). Images was rated independently by the two reviewers according to the guidelines of European Society of Urogenital Radiology (ESUR) [37]. The T_2_WI, DWI and DCE images were scored (1-5) using the Prostate Imaging and Reporting and Data System (PI-RADS) v2 [2], a summed score of all three MR sequences (T_2_WI, DWI, and DCE) was then calculated for the leading lesion in each patient. Additionally, the following imaging characteristics were summarized: 1) tumor location (PZ or TZ); 2) tumor max diameter (Dmax); 3) tumor is MR-visible or not; 4) tumor ADCs; 5) the DCE type; 6) the presence or absence of ECE (MR stage T3a); 7) the presence or absence of SVI (MR stage T3b); 8) the presence or absence of local lymph node (LN) invasion; and 9) the presence or absence of local bone metastasis. Leading lesion boundaries were determined by manually outlining the regions of interest (ROIs) on fusion T_2_WI and high b-value DWI slice-by-slice. Whole-lesion mean ADC value was measured by expending a mono-exponential fitting model. DCE-MRI was classified as three types based on the ESUR guidelines: type 1: slow wash-in and slow wash-out; type 2: fast wash-in and slow wash-out; and type 3: fast wash-in and fast wash-out. The presence of ECE was defined as an infiltration of the peri-prostatic fat, irregular bulging associated with disruption of the capsule, focal thickening, capsular retraction, and peri-capsular spicula. The features of SVI include focal or diffuse low T_2_ SI and/or abnormal contrast enhancement within and/or along the seminal vesicle, restricted diffusion, obliteration of the angle between the base of the prostate and the seminal vesicle, and demonstration of direct tumor extension from the base of the prostate into and around the seminal vesicle (Figure 2). Regarding LN invasion, the LNs over 8 mm in short axis dimension are regarded as suspicious. Nodal groups including common femoral, obturator, external iliac, internal iliac, common iliac, pararectal, presacral, and paracaval, and paraaortic to the level of the aortic bifurcation were evaluated. Local bone metastasis was suspected if focal foci with low SI on T_1_WI, high SI on T_2_WI or DWI on pelvic bone. During the image interpretation, any inter-reader disagreement in classifying MR findings of the lesion was discussed until consensus reached.

### Histopathology

The results of the needle biopsy were recorded from the retrospective database, into which they were entered before RP. All prostate biopsies were obtained using TRUS guidance and were reviewed by dedicated urological pathologists (H.L. and M.B., with 10 years of experience in genitourinary pathology). Each core containing cancer was assigned a primary and a secondary Gleason grade. An overall biopsy Gleason score (GS) was given to each case by identifying the core with the highest Gleason grade. The total number of cores obtained, the number of cores containing cancer, and the percent of biopsy cores involved with cancer (positive for cancer) were recorded. The prostatic specimens after RP were prepared as previously described [16] and examined by the institutional pathology department. The location and extent of invasive tumors were identified and precisely mapped in each section. An overall pathological GS was assigned to the whole gland. Pathological variables included in the study were the tumor number (small tumors with a volume of less than 0.5 cm^3^ were excluded), location, tumor volume and volume percent, primary and secondary GS, cancer invasion on the surgical margin, the presence or absence of ECE, SVI, perineural invasion, and the local LN invasion. Pathological stage of the cancer was assessed according to the 2005 International Society of Urological Pathology Modified Gleason Grading System [38].

### Follow-up

The follow-up included measurements of serum PSA level and a digital examination of rectum (DRE) every 3 months for the first year after RP, at 6-month intervals for the next 2 years, and annually thereafter. In accordance with the European Association of Urology (EAU) guidelines for PCa, disease progression was defined as a serum PSA level that failed to decrease to undetectable levels after surgery, or an undetectable PSA level after surgery with a subsequent detectable PSA level that increased on two or more laboratory determinations, or secondary therapy or clinical recurrence [2]. In this study, a detectable level of PSA (i.e., ≥ 0.2 ng/mL) after surgery was defined as BCR.

### Model development

To predict the probabilities of PCa BCR associated with the variables, firstly, we constructed two risk predictive models based on SVM and multivariate LR analysis. Patient age (< 60 ys and ≥ 60 ys of age) and seven MR imaging parameters, including the PI-RADS score, tumor location (PZ or TZ), tumor max diameter (Dmax), MR-visible or not, tumor ADCs, tumor DCE type (type 1+2 or type 3), and tumor MR T-stage (< T3a, T3a, or > T3a), were included as input data for SVM and LR model. We did not include LN invasion and bone metastasis as the predictive variables because we observed a very few cases with lymph node metastasis (*n* = 3) and bone metastasis (*n* = 0) in this cohort of patients. Secondly, a SVM model based on a popularized D'Amico risk scheme [7] were constructed using the following preoperative clinic-pathologic variables: patient age, the first incorporated preoperative PSA level (< 10 ng/ml, 10-20 ng/ml and > 20 ng/ml), needle-biopsy GS (3+3, 3+4, 4+3, and ≥ 4+4), and DRE and/or TRUS-based clinical TNM stage. In order to investigate whether the addition of MR variables can further improve the performance ability of the D'Amico model in predicting PCa BCR, a modified D'Amico model was constructed by adding the MR variables.

For the multiple LR model, we determined probabilities (Pi) of PCa BCR by using a backward stepwise approach as follows:

[Eq.1] $$Pi=\frac{1}{1+e^{-k}}$$

[Eq.2] $$k=\sum_{1}^{i}BiXi$$

where Xi is the predictive variables and Bi is the regression coefficients determined by the multiple logistic regression analysis.

SVM is a supervised machine learning technique used for classification and regression analysis. SVM algorithm tries to construct an optimal separating hyperplane that maximizes the margin, where the margin is the largest distance to the nearest training data point of any class. Unlike the traditional artificial neural networks (ANNs), the SVM does not have to undertake a trial and error parameter decision process, while determines optimal performance conditions automatically if the kernel type is set. In this study, SVM with radial basis function (RBF) kernel was applied to resolve the two class problems, BCR or BCR-free. A RBF kernel, K, maps the original data with the kernel function as:

[eq.3] $$\text{K}\left(x\right)=exp\left(-g||x-t|{|}^{2}\right)$$

where *x* and t are two feature vectors, and Gamma (g) controls the shape of the decision hyperplane. As in this relatively small patient groups, SVM models were developed and validated using a five-fold cross-validation method that maintained the best compromise between computational cost and reliable estimates. The dataset was randomly divided into 5 mutually exclusively subsets of approximately equal size (*n* = 41), in which 4 subsets were used as training set, and the last subset was used as validation set. The sensitivity and specificity values were calculated for each test fold on the trained model of the other four folds. The performance of each iteration was calculated as the average of the performance values of these five folds. This procedure was repeated for five times, so each subset was used once for validation. As the direct output value of the SVM does not show probabilities of PCa BCR, we converted their output values to the probabilities (*Pi*) by applying a sigmoid function as follows:

[eq.4] $$Pi=\frac{1}{1+e^{-x}}$$

where *x* is the output value of SVM analysis. The value of *Pi* indicates the probabilities that the patient has PCa BCR.

### Statistical analysis

The performance of the LR, SVM, D'Amico and modified D'Amico model was evaluated using a receiver operating curve (ROC) analysis to predict the probabilities of PCa BCR. The ROC curves were estimated using a MedCalc statistical software (version 8.2.0.1, MedCalc Software, Mariakerke, Belgium). The performance parameters including the areas under the ROC curve (Az), sensitivity (SEN), Specificity (SPE) and accuracy (ACC) were reported. Pairwise comparison of the ROC curves was performed. Predicted Kaplan-Meier curves from four nomograms were created and compared to patients' true Kaplan-Meier curves using a Kaplan-Meier analysis by SPSS (Version 22.0, Chicago, IL, USA). A multivariable Cox regression analysis was recruited to determine the independent indictors of PCa BCR, and hazard ratio (HR) was reported. A p value less than 0.05 was considered to indicate a statistically significant difference.
